# Factors affecting domestic tourists’ repeat purchase intention towards accommodation in Malaysia

**DOI:** 10.3389/fpsyg.2022.1056098

**Published:** 2023-03-22

**Authors:** Charles Ramendran SPR, Hui Nee Au Yong, Maryam Kalhoro, Kamarulzaman Bin Mohd Dahari, Farhan Bashir Shaikh

**Affiliations:** ^1^Faculty of Business and Finance, Universiti Tunku Abdul Rahman, Kampar, Perak, Malaysia; ^2^Department of Business Administration, University of Sindh, Jamshoro, Pakistan; ^3^Faculty of Information and Communication Technology, Universiti Tunku Abdul Rahman, Kampar, Perak, Malaysia

**Keywords:** repeat purchase intention, perceived health risk, service quality, sales promotion, domestic tourist’s accommodation, Malaysia

## Abstract

The hospitality and tourist industries depend on homestays and accommodations. Different factors, i.e., promotion strategies, service quality, cleanliness, and perceived health risks, influence tourists’ repeat purchase intention for accommodation. This study highlights different variables influencing domestic tourists’ accommodation repurchase decisions in Malaysia. Around 304 respondents from this quantitative survey are tourists who have already experienced choosing their accommodations during travel. Statistical Packages of Social science software (SPSS-23.0) were used to examine the data through multiple regression and descriptive approaches. The finding suggests that promotion, and services, can positively affect visitors’ purchasing decisions. However, there is a negative but statistically significant link between perceived health risk and repurchase decisions. Furthermore, the result revealed that the predictors of purchase decisions significantly influence selection. This study observed the undervalued quality of services and facilities provided by the hotel industry. The results identified that sustainable practices could enhance the impressive recovery of the tourism industry during and after the pandemic. Furthermore, cleanliness and cost are vital to be considered a primary quality service factor, reducing the perceived health risk, even if there is a pandemic. The study suggested that lodging providers could upgrade on-site facilities and acquire better promotion strategies. The study’s conclusions can increase satisfaction to avoid health risks in any circumstance and promote tourism.

## 1. Introduction

Malaysia’s tourism industry focuses on improving its competitiveness and dynamic environmental sustainability. The provision of sustainable facilitative tourism comes under the United Nations’ sustainable development objectives and the 11th Malaysian Plan (11MP). Around RM 86.10 billion was accumulated from tourist receipts for 26.10 million from inter-state tourism in 2019. Moreover, 4.33 million tourists visited, and industry earnings were reduced to RM 12.7 billion when counting tourist receipts in 2021 (Abumandil et al., 2022). The novel coronavirus strain has substantially impacted tourism and travel behavior (Farzanegan et al., 2021). Tourism remained one of the top features to spread the illness and has been affected along with the supply and value chains during the pandemic (Foo et al., 2020; World Health Organisation [WHO], 2020). A sudden downfall of the global hospitality sector (companies, hotels, restaurants, theme parks, event planners, and booking agencies) was observed (Uğur and Akbıyık, 2020). Presently the tourism industry needed several strategies to recover from the year 2022 and tried to remove the fear of the public by providing more facilities for higher earnings (Halme, 2021). Several factors need to be analyzed to increase domestic tourism by knowing the consumers’ choice of accommodations within Malaysia.

There still needs to be more studies on how consumers’ impressions of service quality, promotional strategies, perceived health risks, and repurchase intention for hotel accommodations among domestic travelers relate to the hospitality business. Rarely are the findings of the expanding literature on purchase decisions placed in a pandemic setting. Most previous studies only examined one of these crucial factors. Therefore, the current study uses this knowledge gap to examine five factors related to purchase decisions.

**Figure 1:**
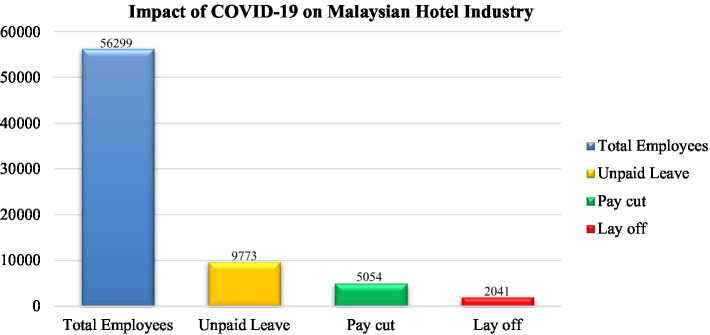
Impact of COVID-19 on the Malaysian Hotel Industry. The Star (2020).

According to reports, the hotel business had an income loss of RM560.72 million during the movement control order (MCO) (Malaysian Association of Hotels [MAH], 2020). Figure 1 shows that out of a 56,299 sample, 2041 workers were laid off from the hotel business, 9,773 (17%) had taken unpaid leave, and 5,054 (9%) had their earnings slashed (The Star, 2020). On the other hand, Kuala Lumpur was severely affected, as 3,641 workers were on unpaid leave, three were fired, 17,826 former workers, and 2,880 (16%) had their salaries reduced (The Star, 2020).

**Figure 2:**
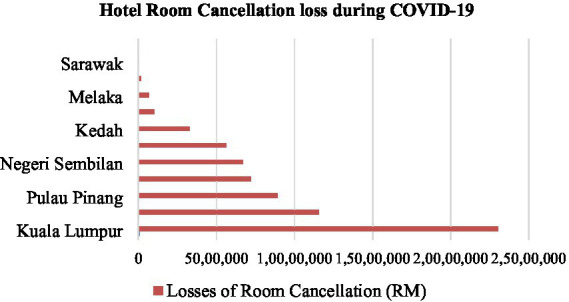
Hotel room cancellations loss. Malaysian Association of Hotels [MAH] (2020) (Ministry of Finance, 2020).

The local hotel business lost RM 75.69 million between January and March 2020. Nevertheless, there has been a dramatic increase in cancellations of reservations in Kuala Lumpur, with 61,859, which costs RM24.91 million in lost income (MAH,2020). Sabah has sustained the second-highest losses after Kuala Lumpur. Arround33,769 cancellations had been made since March 2020, costing RM11.79 million (Shah K. et al., 2020; Shah A. U. M. et al., 2020; Figure 2).

**Figure 3:**
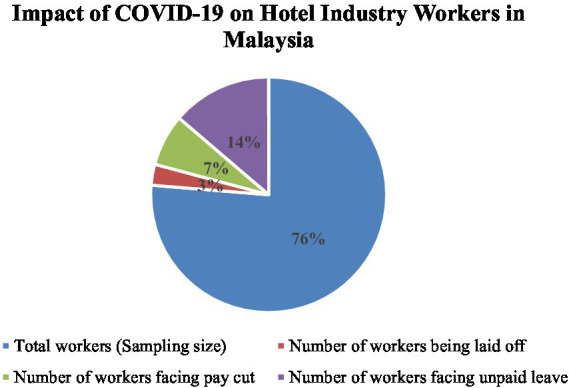
Hotel industry workers during COVID-19 pandemic. Malaysian Association of Hotels [MAH] (2020).

After COVID-19 arose, a total of 17,500,085 hotel reservation cancellations resulted in a loss of RM 68190364. As a result, 76% of the total workers were affected. At the same time, workers without pay leave were 14%. However, hotel industry workers’ cut-off salaries were 7%. Likewise, Laid-off workers were 3% as shown in Figure 3.

The government introduced a wage subsidy scheme by paying RM600 per month for each employee retained for 3 months to offset the growing unemployment risk during COVID-19. However, the industry needs reforms and marketing strategies to solve this issue during the present recovery phase. Different scholars and think tanks offered this threat of COVID-19 as an opportunity for the tourism sector. The elements that affect consumer decision-making are crucial to understanding as the hotel business is directly and significantly affected by consumer behavior during an epidemic. COVID-19 emerged with many factors, i.e., non-refundable income, new travel behaviors, online travel business due to increased internet usage and online banking, the web presence of hotels and tourism directories, and many others (Kaushal and Srivastava, 2021).

The repurchase decision is a significant part of consumer behavior, and ideas date back to the middle of the 1960s. However, several other factors influence client repurchase decisions, i.e., service quality, cleanliness facilities, and reduction in perceived health risk (McKenzie et al., 2011; Vlontzos and Duquenne, 2014; Amanah and Harahap, 2018).

Most of the research was conducted to see natural casualties due to the pandemic crises (Dinçer and Alrawadieh, 2017; Sao et al., 2017; Jeaheng et al., 2020; Raja Omar et al., 2020). In these situations, such as in various financial and health crises, consumers’ judgments of pricing, quality, cleanliness, location, and amenities fluctuate significantly depending on the crisis (McKenzie et al., 2011; Vlontzos and Duquenne, 2014). Consumers repurchase intention and buying habits changed due to increased sensitivity to issues brought on by concerns like job security (Hampson and McGoldrick, 2013). This study will also observe whether factors such as pricing, cleanliness, location, services, and facilities alter the purchase behavior under normal circumstances than crises.

This study is pertinent to domestic tourists choosing lodgings in Malaysia, particularly when prioritizing their selections among the various lodging options offered by the hospitality sector. Tourists increasingly consider hotel attributes, including cleanliness, location, services, perceived health risks to enhance buying choices (Jiang and Wen, 2020). Domestic guests must thus comprehend diverse hotel characteristics and how they affect buying selections. The theory of quality service (Oliver, 1980) states that an increase in quality could surpass performance expectations. As a result, the standard by which the evaluation of service quality is based on consumer expectations. The current study aims to pinpoint the significant determinants of domestic travelers’ accommodation choices influenced by the fear of COVID-19 when travelling in Malaysia’s several states. Based on the above, we present the following research questions:

RQ1. Does service quality influence domestic tourists’ accommodation repurchase intentions?
RQ2. Does the price affect domestic tourists’ accommodation repurchase intentions?
RQ3. Does perceived health risk influence domestic tourists’ accommodation repurchase intentions?
RQ4. Does an increase in service quality reduce the perceived health risk and positively influence domestic tourists’ accommodation repurchase intentions?
RQ5. Does Gender moderate the relationship between perceived health risks and determine domestic tourists repurchase intentions?

## 2. Theoretical framework and hypotheses development

Service quality is the discrepancy between customers’ expectations for service performance before the service contact and their service evaluations. Perceived quality decreases when performance falls short of expectations (Oliver, 1980). The service quality is the predicted performance compared with the actual performance (Grönroos, 1984). Since Gronroos published the first model for assessing service quality, the Nordic model in the early 1980s stated that service performance is received when the functional qualities are a subjective perception of the service delivered. After that, Parasuraman et al. (1985) presented a new model called SERVQUAL, the most well-known and widely used model in the field of service quality. They initially proposed ten dimensions for their model, but in a subsequent version released in 1988, they decreased the dimensions to five. The foundation of this concept was to use five recommended characteristics to measure the gaps between customer expectations and perceptions of the service supplied. Researchers introduced other models and measurements for service quality during these three decades. Therefore, the current research fascinates the service quality theory in explaining the relationship between price, promotion, service, facilities, cleanliness, and purchasing decisions among domestic travelers regarding their selection of accommodation affected by the Covid-19 pandemic while travelling Malaysia.

This study has adopted the variable of intention from the theory of planned behavior TPB (Ajzen, 1985). Several studies in different contexts (i.e., hotel industry, hospitals, educational institutes, and transportation) have used TPB theory to predict the attitude and behavior of customers.

Many empirical studies conducted by different academicians locally and internationally on the impact of promotional strategies, service quality, and purchasing decisions on firms have yielded varied findings. Some of these are given as evidence in the following section.

### 2.1. Service quality and repurchase intention

Meeting the demands of tourists, especially business travelers and hotel service delivery, is the industry’s top priority. A green hotel tries to provide customers with various services, such as transportation, hygiene food, meeting spaces, individualized services, and a place to sleep (Petrenko et al., 2021). Kim (2013) discovered a connection between good customer service and in-store shopping intentions. Furthermore, Aptaguna and Pitaloka (2016) found that the service’s quality positively impacted the user intention of Go-Ojek. Banjarnahor (2017), who examined the effect of service quality on internet service purchase intention in West Jakarta, provided support for this vital contribution. Researchers Pratiwi and Murwanti (2017) showed the impact of service quality on dealer user intention. Similar conclusions were reached by Alharthey (2019), who found that service quality positively influenced consumers’ intentions to purchase groceries. Perceived service quality is one of the critical elements of online purchasing trust (Wiyadi and Ayuningtyas, 2019). Most online selling platforms foster client confidence and enduring connections by providing high-quality services (Shafiee and Bazargan, 2018). Consumers’ opinions of service quality significantly influence their faith in shops, which helps them embrace online purchasing (Ibrahim and Daniel, 2019). For instance, Lien (2017) conducted an exploratory study to look at the effects of stickiness and satisfaction on usage intention and the effects of service quality (environment quality, interaction quality, and result quality) on user pleasure on WeChat. The study concluded that service quality positively affects usage intentions based on data from 310 respondents in China. Setiawan and Sayuti (2017) argued that increasing service quality would encourage client satisfaction and purchases.

The significance of a travel pricing package is now beyond controversy. Tourists enjoy the services and the price since they distinguish the goods (Aguiló et al., 2001). All managers in the tourist industry should understand the pricing structure and be aware of the aspects that impact the pricing policy to achieve more successful package design and service provision. Given that it is one aspect that affects consumer purchase decisions, price plays a crucial part in how customers view a product. Ferdinand et al. (2002) asserts that pricing can influence consumers’ purchasing decisions, which is a significant marketing component. A consumer’s impression of a product’s price significantly impacts whether they decide to buy it (Kotler and Armstrong, 1996). The information about a product is explained and given purpose by consumers’ opinions of the price (Kotler and Keller, 2016).

Customers can assess the hotel’s overall cleanliness by observing guest spaces (such as the lobby, restrooms, rooms, and restaurants), staff spaces, and the personnel’s hygiene (such as uniforms, hands, and heads) (e.g., computers, desks, and chairs). In-service places utilised by a range of consumers, like hotels, require effective hygiene management since these circumstances significantly impact how customers behave and make decisions. In times of a public health emergency, like the present COVID-19 issue, it is imperative to concentrate on general hotel cleanliness and employee hygiene (Faulkner et al., 2021).

Consumer evaluations of service quality and individual attitudes toward decision-making are correlated (O’Neill and Palmer, 2001). Professional services may adjust to consumer characteristics, leading to delighted clients (Jaakkola, 2007). Consequently, the following hypothesis is developed:

*H1*: There is a significant and positive influence of service quality on repurchase intention for domestic tourists’ hotel accommodation selection.

### 2.2. Sales promotion and repurchase intention

It might be challenging to promote travel and tourist sites. Increasing customer awareness while utilizing conventional technologies and sales communication techniques is complex. Regardless of the season, hotels constantly fascinate new consumers due to the fierce rivalry in the hospitality business. As a result, hotels invest substantial money in strategic advertising each year. Print and visual media are essential to promoting accommodation and tourism services since they educate and promote the sale of specialized services. A rising number of hotels and vacation places are luring potential visitors with print, online, and television photos since a picture is worth a thousand words (Warta, T., 2011). The tourism industry consumes the most printed materials (brochures, flyers, folios, and catalogues). Print and visual media are materials whose production and distribution costs are included in the marketing budget to educate existing and potential customers and boost demand for the goods and services provided (Răvar, 2011). Sales promotions are widely used by businesses to expand into new areas, create a strong brand, raise awareness, increase sales, provide value to their products and services, and differentiate themselves from their rivals. According to Köksal et al. (2014), sales promotions strongly impact brand preference and purchase intent. Blythe et al. (2005) claims that sales promotion is essential to brand recognition, which may affect a consumer’s choice to purchase in the future. Additionally, recent research has shown that promotional offers significantly impact a customer’s purchase decision (Biswas and Bhatnagar, 2013). Another hypothesis for this study is provided as follows:

*H2*: Sales promotion positively affects the Repurchase intention for domestic tourists’ hotel accommodation selection.

### 2.3. Perceived health risk and repurchase intention

According to the notion of perceived risk in consumer research, consumers consider risk while making decisions since there is ambiguity about potential harmful outcomes. As a result, perceived risk equals the likelihood that repercussions will materialize times the unfavorable effects of choosing the wrong brand (Mitchell, 1992). The unpleasant repercussions of unforeseen and unknown goods purchases are the source of perceived risk (Bauer, 1960; Rehman et al., 2020). Conceptually, perceived risk and perceived uncertainty are closely related.

Both ideas are viewed as one construct by a corpus of research; perceived risk is a consumer’s sense of unpredictability (Bearden and Shimp, 1982). However, another study focuses on the differences between them; risk perception is made up of two elements: uncertainty and unfavorable outcomes of purchasing a good or service (Mitchell and Vassos, 1998; Rehman et al., 2020). According to the current study, clients perceive risk differently depending on how much ambiguity they have about a product or service.

The notion of perceived risk has been used to explain consumer decision-making practices since the 1960s (Buratti and Allwood, 2019). Since the 1990s, there has been an increase in a study in the hospitality and tourism fields that looks at how visitors perceive risk, what variables affect that perception, and how that perception affects how they travel and make decisions (e.g., Lepp and Gibson, 2003; Lepp et al., 2011; Adam, 2015). Finding various travel-related dangers has been the focus of earlier studies (Yang and Nair, 2014). Travel groups were initially divided into categories according to physical-equipment risk, vacation risk, and destination risk by Fuchs and Reichel (2011).

Reisinger and Mavondo (2005) investigated how the danger of terrorism, the risk to one’s health and finances, and the risk to one’s sociocultural identity affected travel intention. Adam (2015) most recently categorized the perceived risks faced by travelers into six categories: expectations risk, sociopsychological risk, political risk, financial risk, and environmental risk. Health risk is one of several categories of a perceived risk that refers to how tourists or consumers of hospitality businesses perceive the danger to their physical health as a result of uncontrollable events like terrorism, political unrest, natural catastrophes, and pandemics.

In the past, research on health risks has aimed chiefly to comprehend how visitors perceive health risks when they partake in risky activities in contexts related to adventure tourism (e.g., Bentley and Page, 2008; Buckley, 2012). After a string of incidents, including the 9/11 terrorist attacks in 2001, the SARS epidemic in 2003, the Bali bombings in 2002, and the Asian tsunami in 2004 (Yang and Nair, 2014; Williams and Baláž, 2015), a growing corpus of research started to focus on concerns related to health or safety threats. Travelers now perceive a significant health risk when they visit locations or hospitality facilities due to the COVID-19 epidemic.

Because of health concerns, most visitors will still be hesitant to travel even after the epidemic; therefore, risk-reduction tactics must be implemented by those working in the hospitality and tourism industries. As previously stated, perceived risk results from uncertainty. Thus, one of the most critical aspects of health risk management is effectively decreasing uncertainty. In this context, epistemic and aleatory uncertainty offers fundamental knowledge to comprehend perceived health risks. First, ignorance leads to epistemic uncertainty, also known as internal, functional, subjective, or reducible uncertainty (Yoe, 2019).

Additional product or service knowledge might lessen perceived risk and epistemic uncertainty. Broader marketing research has primarily concentrated on risks of epistemic uncertainty, such as financial risk (failure to meet consumers’ financial needs), psychological risk (damage to consumers’ sense of self-worth), performance risk (failure to provide benefits to customers), social risk (loss of customers’ social status), time risk (failure to perform on time), and satisfaction risk (failure to be satisfied with the performance of products or services).

On the other hand, aleatory ambiguity is strongly correlated with perceived health risk. Aleatory uncertainty results from the unpredictability and innate variability of the physical universe. Aleatory uncertainty is strongly correlated with unforeseen hazards at the destination level in the hospitality and tourist sectors, such as perceived health risks (Bruce, 2002). Therefore, there are certain restrictions on how much aleatory uncertainty may be reduced by gathering more data (Yoe, 2019).

Earlier tourism and hospitality research has identified several tourism risks associated with asymmetrical uncertainty, including operational risk (the possibility of mechanical, equipment, or organizational problems), health risk (the possibility of becoming ill or contracting certain diseases), physical risk (the possibility of physical danger or injury), political risk (the possibility of being caught up in a political upheaval), and crisis risk (the possibility of natural disasters). One is perceived health risk, which relies heavily on aleatory uncertainty and cannot be reduced by learning more facts.

One additional critical factor for health risk perception is cleanliness. The hospitality and tourism industry needs to provide a more extensive level of cleanliness to attract customers. In particular, hotel cleanliness is a critical aspect of hotel firms’ success; most hotel customers are highly concerned with the quality of hotel cleaning (Barker, 2015). Hotel cleanliness has been examined in terms of its impact on customer satisfaction (Liu and Jang, 2009), service quality (Barber et al., 2011), and hotel security (Amblee, 2015). Bitner (1992) argued that the perceived services cape (physical settings) elicits cognitive and emotional responses. In this regard, how hotel customers expect the cleanliness of hotel physical environments influences their perceived health risk during the COVID-19 pandemic. Innovative services in hotel cleaning systems can allow tourists to develop a certain level of expected cleanliness (Zemke et al., 2015; Zeng et al., 2020). When prospective customers know that enhanced cleaning technology systems are implemented at hotels, they are likely to have higher levels of expected cleanliness, resulting in lower health risk levels (Zemke et al., 2015). Thus, the following hypothesis is proposed.

*H3*: Expected service quality of the hotel will affect perceived health risk, such that hotel customers will perceive lower levels of health risk which significantly affect the repurchase intention.

*H4*: Perceived health risk is negatively associated with repurchase intention for domestic tourists’ hotel accommodation selection.

### 2.4. Hypothesized research model

**Figure 4:**
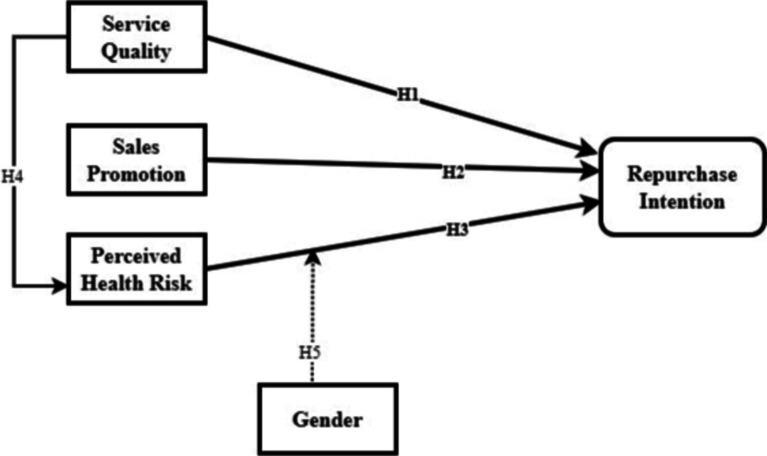
Hypothesized research model.

The present study has proposed the research framework and the research hypothesis tested in the study. Service quality, promotion, perceived health risk directly and significantly influences the domestic tourists repurchase decision for the accommodation during their trip during the period of COVID-19 in Malaysia (refer to Figure 4).

## 3. Materials and methods

The quantitative method was used in the investigation. Given that the respondents are likely bilingual, this study’s survey questionnaires were created in Malay and English. The questionnaire’s first section covers socio-demographic factors. Other portions include buying price, service, promotional strategies, environment, and cleanliness. Questionnaires will be distributed to gather primary data, with the individual (domestic passengers in Malaysia) serving as the analytic unit. The researcher will gather the data between January and March 2022. Due to the popularity of these states as tourist destinations in Malaysia, this study concentrated on domestic tourists from Kuala Lumpur, Perak, Selangor, Pahang, Sabah, Negeri Sembilan, Sarawak, Melaka, Penang, Johor, Terengganu, and Kelantan (Farhana et al., 2020). The respondents received the most significant guarantees, assuring them that throughout the study, all information provided was always treated with utmost secrecy and respect. The email was used to administer the surveys individually (Dobbs et al., 2011). The minimal sample size for this study is determined using a power analysis.

The sample parameters were selected using the G*Power 3.1 program (Faul et al., 2007, 2009). It was determined that 138 is the minimal sample size to predict the results. Thus, a regression-based model must be tested using more than 138 samples (Cohen, 1992; Faul et al., 2007, 2009). More sample size is adequate; therefore, a convenient sampling technique was utilized to choose the appropriate sample size for this investigation (304 domestic tourists).

### 3.1. Measures

The measures used in this study and their operational definitions were covered in this part. Each respondent was asked to rate their level of agreement with each purchase choice statement on a five-point scale, with 1 denoting a “strongly disagree” and 5 indicating a strongly agree.

#### 3.1.1. Repurchase intention

A six-item measure created by Kotler and Keller (2012) has been modified for this investigation. The instrument’s internal consistency was considered satisfactory in prior investigations, with Cronbach alphas ranging from 0.90 to 0.96.

#### 3.1.2. Service quality

The modified 4-item instrument adopted from the study (Zietsman et al., 2018). The instrument’s internal consistency was considered satisfactory in prior investigations, with Cronbach alphas varying 0.70–0.96.

#### 3.1.3. Sales promotion

The research modified a six-item questionnaire Chandon et al. (2000) created. The instrument’s internal consistency was considered satisfactory in prior investigations, with Cronbach alphas varying 0.53 to 0.78.

#### 3.1.4. Perceived health risk

For this study, Shin and Kang (2020), 4 elements have been modified. The instrument’s internal consistency was considered satisfactory in prior investigations, with Cronbach alphas varying from 0.83 to 0.70. Table 1 displays the operationalization.

**Table 1:** Measures of the constructs.

	Operational definition	Items adapted	Reference
Repurchase intention	Financial economics, technology, politics, culture, products, pricing, location, advertising, tangible evidence, people, and procedures impact consumer decisions.	I chose this hotel because of its outstanding quality.	Kotler and Keller (2012)
		I chose this hotel because of the high level of service.	
		I choose this lodging because of word-of-mouth advertising (WOM).	
		I chose this lodging based on my own experience.	
		I chose this hotel because of its reputation.	
Service quality	All the benefits that a consumer receives from using or owning a product.	This hotel has reasonable pricing.	Beneke and Zimmerman (2014)
		This hotel’s cost is fair for the level of service it provides.	
		The cost of this lodging is reasonable.	
	The discrepancy between a customer’s expectation of service and what they receive.	The result of this hotel service meets my chosen needs.	Zietsman et al. (2018)
		The length of this hotel’s service is in keeping with the level I liked.	
		According to my preferences, the hotel’s product is decent and nutritious.	
		This hotel’s services and products have a high reputation.	
Promotion	A range of incentive techniques, most of which are short-term, are used to encourage buyers or sellers to make purchases faster or to make more sales.	If this lodging provider has an alluring promotion, I will use it.	Chandon et al. (2000)
		This lodging establishment offers promotions.	
		They force me to buy from them out of the blue because of their promotion effort.	
		I want to be more inclined to use this lodging service regularly.	
		I am tempted to make impulsive purchases at this hotel.	
		This hotel serves as a constant reminder for me to use its services.	
Perceived health risk	A key factor that influences tourists’ decision-making processes for repurchase intention; they are less likely to visit a destination when they perceive high levels of health risk at the destination.	I feel nervous about visiting hotel because of health concerns.	Shin and Kang, 2020
		Visiting hotel is a risky decision for my health.	
		There is high probability that visiting hotel would lead to a health problem.	
		I feel uncomfortable visiting hotel because of my health safety.	

## 4. Data analysis and results

Data were filtered, reviewed, and validated to analyze descriptive statistics before entering the statistical package for social science (SPSS) version 23. The demographic statistics show that among the 304 respondents, only 190 (62.5 percent) were men, and 114 (37.5 percent) were women. There were 164 persons in the 25–35 age range (53.9 percent of the total population) and 118 in the 46–55 age range (38.8 percent). Those aged 56 to 65 made up 22 (7.2 percent) of the population. Regarding marital status, 170 respondents (or 55.9 percent) made up the single group, while 134 respondents (44 percent) were married. Only 43 respondents (14.1 percent), according to the respondents’ educational profiles, have completed the SPM. Following this are those with a diploma, with 91 (29.9 percent), and those with a bachelor’s are 130, (42.7 percent). Master’s degree credential was held by roughly 66 (21.1 percent) respondents. Finally, 17 (5.5 percent) of the responders were PhD holders. In terms of race, 228 (75 percent) of the respondents were Malays, 53 (17.4 percent) were Chinese, 17 (5.5%) were Indians, and 6 (1.9 percent) were from other races. In terms of wages, there were 68 (22.3 percent) people earning less than RM2,000, 158 (51.9 percent) people earning more than RM4,000, and 56 people earning between RM2,001 and RM3,000 (18.4 percent). Finally, there were 22 people in the RM3,000–RM4,000 bracket (7.2 percent). Regression analysis was performed using the SPSS programs. To measure the moderation hypothesis, we employ the methods outlined by Aguinis (2004).

### 4.1. Reliability

The reliability statistics of all the variables, i.e., RI, SQ, PRO and HR are 0.873, 0.852, 0.874, and 0.929 are more significant than the standard value of 0.70 as shown in Table 1. The reliability values for Cronbach’s Alpha should be better than 0.70 (Flynn et al., 1990).

### 4.2. Exploratory factor analysis

Additionally, the validity is distinguished when the main components are computed using factor analysis while carrying out the Varimax rotation. According to Hair et al. (2006), the minimal factor loading requirements vary from 0.50 to 0.80; loadings of 0.50 or more are considered more important. Hair et al. (2010) highlighted the significance of the KMO value of 0.90 in related research. A value of 0.80 is considered excellent, ordinary as 0.70, inadequate as 0.60, bearable but unsatisfactory as 0.50, and horrible as less than 0.50.

Four components from an Explanatory Factor Analysis (EFA) were used to study the repurchase intention. The factor loadings of five repurchase intention criteria are shown in Table 2. The KMO value should be in the 0–1 range. When every variable is completely and accurately predictable by every other variable, the KMO value is closer to 1 (Hair et al., 2010). Bartlett’s test results were also extremely significant (*p* = 0.00). Results from the MSA test for individual measurements indicate that the values are within acceptable bounds. Each item’s MSA value was set to be higher than 0.50. It shows that the requirements for factor analysis have been satisfied. The results show that the KMO and Chi-Square values and the research items met the requirements for factor analysis. The outcomes are low factor loading (0.50) or double loading. Results for all the products’ loadings range from 0.509 to 0.642.

**Table 2:** Reliability result.

Variables	No. of items	Cronbach’s alpha (*α*)
Repurchase intention (RI)	10	0.873
Service quality (SQ)	4	0.852
Promotion (PRO)	6	0.874
Perceived health risk (HR)	4	0.929

The KMO measures of repurchase decisions are 0.689, the Chi-square value is (117.389), and the significance level is 0.000 as shown in Table 3. The KMO and chi-square test results show that all the used items met the requirements for factor analysis and are thus suitable for repurchase intention. After each phase of the approach, the factor loading of four pricing item components indicates either low factor loadings (0.50) or double loading, as indicated in the table. Every item received a score of 0.701 and 0.857.

**Table 3:** Exploratory factor analysis for purchasing decision.

Items	Factor loadings	Kaiser-Meyer-Olkin measure of sampling adequacy	Bartlett’s test of sphericity	Df	Sig
			Approximation of chi-square		
*Purchasing decision*					
RI1	0.623	0.689	117.389	15	0.00
RI2	0.573				
RI3	0.509				
RI4	0.515				
RI5	0.642				
RI6	0.542				
RI7	0.701				
RI8	0.758				
RI9	0.857				
RI10	0.738				
*Service quality*					
SQ1	0.861	0.800	319.666	6	0.00
SQ2	0.812				
SQ3	0.819				
SQ4	0.844				
*Promotion*					
PRO1	0.718	0.867	557.511	15	0.00
PRO2	0.644				
PRO3	0.771				
PRO4	0.856				
PRO5	0.834				
PRO6	0.871				
*Perceived health risk*					
HR1	0.913	0.872	803.271	10	0.00
HR2	0.911				
HR3	0.874				
HR4	0.856				

Typically, the KMO (total items) value is at least 0.50 Coakes and Steed (2010) and Hair et al. (2006). The findings of Bartlett’s test were similarly very significant (p = 0.00). Individual measures’ MSA test results show that the levels are within permissible limits. The MSA value for each item was set to be greater than 0.50. The ten service quality items examined at each stage of the process were shown in Table 3, with either a low factor loading (less than 0.05) or double loading. The loadings were between 0.81 and 0.86. Table 3 also showed the six items of sales promotion items that were examined at each stage of the operation, showing either a double loading or a low factor loading (less than 0.05). The loadings were between 0.64 to 0.871. The loadings for the four perceived health risk factor varied from 0.874 to 0.913. It demonstrates that the prerequisites for factor analysis have been met.

### 4.3. Hypotheses testing

Multiple regression analysis is used to identify the answers to the research questions and test the research hypothesis using varied outcomes (Table 4).

**Table 4:** Hypotheses testing.

Hypotheses	Standardized coefficients beta	*t*-value	Sig.	Decision
(Constant)		5.026	0.000	
H1: Service quality → Repurchase intention	0.161	2.031	0.044	Supported
H2: Sales promotion → Repurchase intention	0.284	4.565	0.000	Supported
H3: Perceived health risk → Purchase decision	−0.146	−2.446	0.015	Supported
H4: Service quality → Perceived health risk → Repurchase intention	0.181	2.743	0.032	Supported
*R*^2^ 0.691	*F* = 33.398	Sig. = 0.000	DF = 5	

The hypothesis testing was performed by using regression analysis. Table 3 represents the results of the analysis. The repurchase intention, service quality, promotion, and perceived health risks (*R*^2^) *R* square was 0.463 with an adjacent *R* square (*R*^2^) of 0.463 and an *F* value of 33.398. The result suggests that each of the four independent factors can account for 69.1 percent of the variation in purchasing decisions. The study demonstrates a significant positive relationship between service quality and repurchase intention *β* = 0.161 at *p* < 0.044. There is a significant positive relationship between promotion and repurchase intention, i.e., *β* = 0.284 at *p* < 0.000. There is a significant negative relationship between facilities and purchasing decision, i.e., *β* = −146 (*p* < 0. 015). The perceived health risks mediate the relationship between service quality and repurchase intention was significant *β* = 181 at *p* < 0. 032.

#### 4.3.1. Moderation effect

According to the study in the previous section, service quality and marketing tactics can, to some extent, forecast customers’ intentions to make another purchase. Repurchase intention is inversely correlated with perceived health risk. On the other hand, we need to make sure that gender moderating has an impact on this relationship.

Aguinis (2004) outlined the procedures for moderate regression analysis as follows:

1. Centralize variables, which entails deducting their means from the newly collected data so that the mean is 0. This will help the regression equation’s multicollinearity issue between variables.
2. Construct interactive items. It centralizes the moderating variable and multiplies the centralized independent variable.
3. Create a model. To calculate the R12 square and R22 square coefficients, independent and dependent variables (centralized values) and interactions are included in the regression equation. Suppose R22 is higher than R12 and Sig. There is a moderating variable and a moderating effect, and the value of the centralized variables is significant (*p* value = 0.05).

The Table 5 displays the moderating impact of Gender on perceived health risk and repurchase intention. It is concluded that Gender had a significant moderating effect on Perceived Health Risk and Repurchase Intention. As the estimated coefficient changed from 0.635 to 0.643, R22 is higher than R12 by 0.012, and the Significant value of GenderxHR reaches the significant level (*P 0.05*) after the interaction item (GenderxRI) was introduced in the second model. Therefore, hypothesis H5 is accepted by this research.

**Table 5:** Effect of gender on the relationship between HR and RI.

Model		*B*	*R* ^2^	*R*^2^ Change	Beta	*T*	Sig.
1	(Constant)	5.890	0.635	0.635		24.837	0.000
	Gender	−1.140			−0.731	−20.193	0.000
	RI	−0.210			0.206	5.777	0.000
2	(Constant)	5.855	0.647	0.012		24.359	0.000
	Gender	−1.141			−0.731	−20.839	0.000
	RI	0.243			0.239	6.530	0.000
	GenderxRI	−0.150			−0.115	−3.192	0.002

a. Dependent Variable: Perceived Health Risk.

## 5. Discussion

The concerns about health risks within the hotel and tourist industries need study and investigation given the increased perception of destination and hospitality properties as hazardous locations to go to during the COVID-19 pandemic. In both pandemic and post-pandemic settings, this study looked at reducing risk through service provision and promotional strategies, which can affect guests’ perceptions of health risk and intent to return.

This study’s findings show that the accommodation’s effective services (tangible and intangible) are vigilantly favourable to the repurchase decision, consistent with earlier studies (Jayanti and Zuhri, 2017; Ramadhani et al., 2019). While making decisions service quality is crucial when making decisions (Kotler and Keller, 2016). According to the Theory of Service Quality Clients (Oliver, 1980), people will view quality as being low if performance falls short of their expectations. Quality will increase if performance meets or surpasses those expectations. As a result, the standard by which service quality is evaluated is based on consumer expectations—additionally, client satisfaction and the likelihood of using the business increase as service quality increases. The data indicated a good and substantial link between promotions and purchase choices. Numerous organizations have argued that marketing is the most effective way to sway consumers’ purchase decisions (Astuti et al., 2015; Wongleedee, 2015). The underlying premise of consumer promotion is that consumers will be exposed to high-quality, practical items to make wise purchase decisions *via* various consumer promotion instruments.

Additionally, numerous organizations have stated how a person’s internal environment might affect their decision to make a purchase (Nasikan, 2013; Kuspriyono and Nurelasari, 2018). Therefore, hotels or managers need to pay attention to marketing techniques to influence domestic tourists’ lodging choices when visiting Malaysia and how the COVID-19 epidemic may impact those choices. The findings of this study are consistent with earlier research (Ariastini et al., 2017; Hermiyenti and Wardi, 2019). The regression analysis results demonstrated that service quality has a favourable and substantial impact on domestic visitors’ decisions about their choice of lodging when visiting Malaysia and being impacted by the COVID-19 epidemic. The findings by Laksmi et al. (2022), which showed a substantial correlation between service quality and purchase choice, are comparable to this outcome. In their decision-making process, consumers often use service quality as a comparison indicator when choosing from among the offered services. Additionally, comfortable customers who receive good service will be satisfied.

To enhance customers’ perception of their hotels or homestays, hotels and other service providers could incorporate service perceptions and expectations metrics. In the Borobudur Hotel in Jakarta, the relationship between amenities and buying choices significantly impacts customer happiness. The results are consistent with those of Nurcahyo et al. (2017), who found that facilities had a significant and favorable impact on purchase decisions. This result showed no evidence to suggest a link between cleanliness and purchase behavior.

The researchers observed that the client’s purchasing behavior was unaffected by cleanliness. This conclusion is consistent with that of Vilnai-Yavetz and Gilboa (2010), which also revealed no correlation between cleanliness and purchase behavior. The current study adds to the body of knowledge by describing how to approach behaviors influenced by cleanliness. Cleanliness is crucial because poor service performances or failures affect client satisfaction and purchasing decisions more than good service performances (Fu and Mount, 2002; Mattila, 2006). Based on this finding, D’Astous (2000) argues that service managers should concentrate on removing environmental irritants that annoy customers before attempting to enhance other aspects. Therefore, the findings indicated that domestic travelers’ decisions about the type of lodging they would choose in Malaysia during the COVID-19 outbreak were influenced by pricing, service quality, advertisements, and promotional strategies and facilities.

## 6. Implications

This section indicates both managerial and theoretical implications drawn from the results of this study. On the other hand, this study provided significance in terms of theoretical level. In Malaysia, no study emphasized the purchase decision. This study is intended to identify key contributing factors for domestic travelers in their selection of accommodation affected by the COVID-19 pandemic while travelling in Malaysia. Theoretically, the study is underpinned by the theory of service quality, planned behaviour theory and perceived health risks. As a result, consumers’ expectations serve as the benchmark against which service quality is measured.

This study addressed a research vacuum in this area by demonstrating the strong influence of perceived health risks on hotel booking intention. Notably, the study’s findings suggest that consumers’ perceptions of health risks may only sometimes be considered when choosing a hotel. In light of the COVID-19 epidemic, the non-compensatory decision rule can serve as a theoretical framework to comprehend the decision-making practises of visitors or hospitality clients.

Furthermore, customer happiness and intent to utilize the service again improve as service quality improves. According to this idea, the quality of services is determined by comparing expected performance with perceptions of actual performance, as initially prescribed by Grönroos (1984). It has also been proposed that *quality* can be defined as a person’s subjective assessment of a product’s or service’s excellence and perfection. The philosophy of service quality is founded on the literature on product quality and client satisfaction (Brady and Cronin, 2001). In 1988, Zeithaml defined *service quality* as “an assessment of customers from the overall excellence of services”.

Future researchers can use this study as a reference to extend accommodation selection. This study has five proposed factors: price, service quality, promotion, facilities, and cleanliness. This is the first research to include various hotelier industry predictors.

Since customers are familiar with famous hotels and homestay settings and services, it is possible that if some items need to meet customers’ standard of cleanliness, they can quickly notice the point even when they visit for the first time. This section outlines the theoretical and managerial implications of the study’s findings. This study aims to pinpoint the significant influencing elements that domestic traveler should consider while choosing accommodations in Malaysia during the COVID-19 epidemic. The service quality supports the study. As a result, the standard by which service quality is evaluated is based on consumer expectations.

According to this concept, comparing predicted performance with perceptions of actual performance, as initially suggested by Grönroos (1984), determines the quality of services. Additionally, it has been suggested that quality might be described as a person’s subjective evaluation of the excellence and perfection of a good or service. The literature on product quality and customer satisfaction is the foundation for service quality (Brady and Cronin, 2001).

This study can be used as a guide for future studies to broaden their choice of accommodations. Price, service quality, promotion, amenities, and cleanliness are the five recommended elements in this study. This research is the first to consider several factors specific to the hospitality sector in Malaysia during the COVID-19 era. Customers may readily notice if some objects need to reach their level of cleanliness even when they arrive for the first time since they are familiar with well-known hotels’ and homestays’ settings and services.

Additionally, they should be aware that rather than rating individual objects, guests evaluate hotels on various integral aspects. To achieve the best hotel cleanliness, hotel managers must consider various factors. To correctly manage their limited resources and make the most effective use of them, hotel managers must comprehend what customers value most when evaluating the cleanliness of their accommodations. Additionally, the study’s findings suggested that a hotel’s cleanliness affects guests’ satisfaction levels and decisions about returning in the future.

## 7. Future suggestions and limitations

This research has some limitations. The first limitation concerns the sampling frame used. Since this study used convenience sampling, it cannot represent an entire population. In addition, this study chose domestic tourists from Kuala Lumpur, Perak, Selangor, Pahang, Sabah, Negeri Sembilan, Sarawak, Melaka, Penang, Johor, Terengganu, and Kelantan, Malaysia. However, it is hard to generalize the respondents to represent eleven states of Malaysia. The model’s validity across regions of Malaysia may be compared for future studies. A more and higher representative sample should be taken into consideration. A future study could also examine the dimensions of accommodation choice criteria between domestic and international tourists or high versus low-end. Finally, a future study on tourism in mentioned states could focus on destination development and the local perception of the current form of tourism, assessing signs of tourism, and developing tools to manage them in a more sustainable approach.

Additionally, domestic passengers were selected for this survey to serve as a representative sample of Malaysian respondents. However, extrapolating respondents to encompass all Malaysians is challenging. A future study may examine the model’s validity throughout Malaysia’s regions.

A more significant, more representative sample should be considered. This study’s potential future extensions may look at factors like choosing high-end versus low-end accommodations or the differences in accommodation selection criteria for local versus foreign tourists. Finally, future research on tourism can concentrate on destination creation, local perceptions of the existing type of tourism, identifying tourism signals, and creating instruments to manage them more sustainably.

The last restriction is the usage of a Likert scale for significance ratings. When consumers say something significant, they could be using their judgment. Other approaches, such as experimental design and decision modelling, are thus beneficial in reducing this issue. Future studies should keep track of the characteristics that buyers value while making judgments about what to buy. Future research should test across a range of demographic categories, including those based on age and wealth.

In reality, deploying technology to lower health risks is a company-wide process; various technologies may be used throughout hotel facilities, including hotel rooms, lobbies, front offices, restaurants, swimming pools, etc.

The adoptions at several contact points on perceived health risks and hotel effects of more advanced technologies must be examined in future studies.

## 8. Conclusion

This study contributes to the knowledge about hotels for tourists in Malaysia and Southeast Asia. This thesis investigated how the COVID-19 outbreak influenced domestic travelers’ choice of accommodations when visiting Malaysia. It has been discovered that the purchase choice is influenced by how domestic tourists perceive the pricing, service quality, promotion, amenities, and cleanliness. The results agree with other studies regarding how tourists behave while choosing accommodations in Malaysia. Results showed favorable and substantial relationships between pricing, service quality, promotion, and facilities and purchase decisions.

Contrarily, cleanliness could have been more essential and helpful in the choice to buy. This research provides a scholarship for the Malaysian hotel industry’s target audiences and shifting tourist patterns. The findings of this study help guide hotels’ decisions on amenities, customer service, and marketing. Additionally, it will assist hoteliers in developing and maintaining distinctive selling factors. According to research findings, more excellent thought and action are needed regarding infrastructure design and development, particularly regarding municipal parking avenues.

Given the increase in same-day visits, this research has found that such infrastructure issues are predicted to worsen. Additionally, to further divert the tourist crowds, the destination may need to incorporate more lodging alternatives and day activities outside the city Centre, or it may want to explore adopting laws that cap the number of visitors and set reasonable boundaries on change. The report is also an excellent resource for hotel owners looking to grow their companies internationally. This research has important management ramifications for hotels and resorts.

## Data availability statement

The original contributions presented in the study are included in the article/supplementary material, further inquiries can be directed to the corresponding author.

## Author contributions

CSPR highlighted the problem statement, set the hypotheses, and execute the research process. KD was supervising, correcting, and editing the manuscript. HY conceived the whole project idea. MK has processed the data analysis with the help of Smart PLS and SPSS software. FS performed the pilot testing and data collection process. All authors contributed to the article and approved the submitted version.

## Funding

This research was one of the outcomes of the project supported by the Universiti Tunku Abdul Rahman, Kampar, 31900, Perak, Malaysia.

## Conflict of interest

The authors declare that the research was conducted in the absence of any commercial or financial relationships that could be construed as a potential conflict of interest.

## Publisher’s note

All claims expressed in this article are solely those of the authors and do not necessarily represent those of their affiliated organizations, or those of the publisher, the editors and the reviewers. Any product that may be evaluated in this article, or claim that may be made by its manufacturer, is not guaranteed or endorsed by the publisher.

## References

[ref1] Abumandil M. Ekmeil F. A. R. Younus A. M. Alkhawaja M. I. (2022). Mobile augmented reality elements and social media usage on smart tourism in Penang: Malaysian. ECS Trans. 107, 10935–10943. doi: 10.1149/10701.10935ecst

[ref2] Adam I. (2015). Backpackers' risk perceptions and risk reduction strategies in Ghana. Tour. Manag. 49, 99–108. doi: 10.1016/j.tourman.2015.02.016

[ref3] Aguiló P. M. Alegre J. Riera A. (2001). Determinants of the price of German tourist packages on the Island of Mallorca. Tour. Econ. 7, 59–74. doi: 10.5367/000000001101297739

[ref4] Aguinis H. (2004). Regression Analysis for Categorical Moderators. New York Guilford Press.

[ref5] Ajzen I. (1985). “From intentions to actions: a theory of planned behavior” in Action Control (Berlin, Heidelberg: Springer), 11–39.

[ref6] Alharthey D. B. (2019). Impact of service quality on customer trust, purchase intention and store loyalty, mediating role of customer satisfaction on customer trust and purchase intention: study of grocery shopping. Br. J. Market. Stud. 7, 40–61.

[ref7] Amanah D. Harahap D. A. (2018). Examining the effect of product assortment and price discount on online purchase decisions of university students in Indonesia. Jurnal Manajemen Dan Kewirausahaan 20, 99–104.

[ref8] Amblee N. (2015). The impact of cleanliness on customer perceptions of security in hostels: a WOM-based approach. Int. J. Hosp. Manag. 49, 37–39. doi: 10.1016/j.ijhm.2015.04.011

[ref9] Aptaguna A. Pitaloka E. (2016). Pengaruh Kualitas Layanan Dan Harga Terhadap Minat Beli Jasa Go-Jek. Widyakala 3, 49–56.

[ref10] Ariastini N. K. D. Yuniarta G. A. Ak S. E. Si M. Kurniawan P. S. ST M. (2017). Pengaruh Kompetensi Sumber Daya Manusia, Sistem Pengendalian Internal Pemerintah, Proactive Fraud Audit, Dan Whistleblowing System Terhadap Pencegahan Fraud Pada Pengelolaan Dana Bos Se-Kabupaten Klungkung. JIMAT (Jurnal Ilmiah Mahasiswa Akuntansi) Undiksha 8.

[ref11] Astuti R. Silalahi R. L. R. Wijaya G. D. P. (2015). Marketing strategy based on marketing mix influence on purchasing decisions of Malang apple consumers at giant Olympic Garden mall (MOG), Malang city, East Java province, Indonesia. Agric. Agricult. Sci. Proc. 3, 67–71. doi: 10.1016/j.aaspro.2015.01.015

[ref13] Banjarnahor H. (2017). An association with the participative leadership style influences job satisfaction, affective commitment, and continuous head junior high school in Medan. World J. Educat. Res. 4

[ref14] Barber N. Goodman R. J. Goh B. K. (2011). Restaurant consumers repeat patronage: a service quality concern. Int. J. Hosp. Manag. 30, 329–336. doi: 10.1016/j.ijhm.2010.08.008

[ref9003] Barker A. D. (Ed.). (2015). Identity and intercultural exchange in travel and tourism (Vol. 42). Channel View Publications.

[ref15] Bauer R. A. (1960). Consumer behavior as risk taking. In Proceedings of the 43rd National Conference of the American Marketing Association, June 15, 16, 17 Chicago, IL: American Marketing Association.

[ref16] Bearden W. O. Shimp T. A. (1982). The use of extrinsic cues to facilitate product adoption. J. Mark. Res. 19, 229–239. doi: 10.1177/002224378201900207, PMID: 19113951

[ref17] Beneke J. Zimmerman N. (2014). Beyond private label panache: store image and perceived price effect on brand prestige. J. Consum. Mark. doi: 10.1108/JCM-12-2013-0801

[ref18] Bentley T. A. Page S. J. (2008). A decade of injury monitoring in the New Zealand adventure tourism sector: a summary risk analysis. Tour. Manag. 29, 857–869. doi: 10.1016/j.tourman.2007.10.003

[ref19] Biswas S. Bhatnagar J. (2013). Mediator analysis of employee engagement: role of perceived organizational support, PO fit, organizational commitment and job satisfaction. Vikalpa 38, 27–40. doi: 10.1177/0256090920130103

[ref20] Bitner M. J. (1992). Servicescapes: the impact of physical surroundings on customers and employees. J. Mark. 56, 57–71. doi: 10.1177/002224299205600205

[ref21] Blythe A. R. Blythe T. Bloor D. (2005). Electrical Properties of Polymers. New York Cambridge University Press.

[ref22] Brady M. K. Cronin J. J. Jr. (2001). Some new thoughts on conceptualizing perceived service quality: a hierarchical approach. J. Mark. 65, 34–49. doi: 10.1509/jmkg.65.3.34.18334

[ref23] Bruce M. L. (2002). Psychosocial risk factors for depressive disorders in late life. Biol. Psychiatry 52, 175–184. doi: 10.1016/S0006-3223(02)01410-5, PMID: 12182924

[ref24] Buckley R. (2012). Sustainable tourism: research and reality. Ann. Tour. Res. 39, 528–546. doi: 10.1016/j.annals.2012.02.003, PMID: 36160520

[ref25] Buratti S. Allwood C. M. (2019). The effect of knowledge and ignorance assessments on perceived risk. J. Risk Res. 22, 735–748.

[ref26] Chandon P. Wansink B. Laurent G. (2000). A benefit congruency framework of sales promotion effectiveness. J. Mark. 64, 65–81. doi: 10.1509/jmkg.64.4.65.18071

[ref9001] Coakes S. Steed L. M. (2010). Ong. SPSS Version 18.0 Analysis without Anguish.

[ref27] Cohen J. (1992). Statistical power analysis. Curr. Dir. Psychol. Sci. 1, 98–101. doi: 10.1111/1467-8721.ep10768783, PMID: 36622864

[ref9002] D’Astous A. (2000). Irritating aspects of the shopping environment. Journal of Business Research 49, 149–156.

[ref28] Dinçer M. Z. Alrawadieh Z. (2017). Negative word of a mouse in the hotel industry: a content analysis of online reviews on luxury hotels in Jordan. J. Hosp. Market. Manag. 26, 785–804. doi: 10.1080/19368623.2017.1320258

[ref29] Dobbs C. Escobedo F. J. Zipperer W. C. (2011). A framework for developing urban forest ecosystem services and goods indicators. Landsc. Urban Plan. 99, 196–206. doi: 10.1016/j.landurbplan.2010.11.004

[ref30] Farhana Z. Tanni S. A. Shabnam S. Chowdhury S. A. (2020). Secondary education during lockdown situation due to COVID-19 pandemic in Bangladesh: teachers’ response on online classes. J. Educ. Pract. 11, 97–102.

[ref31] Farzanegan M. R. Gholipour H. F. Feizi M. Nunkoo R. Andargoli A. E. (2021). International tourism and outbreak of coronavirus (COVID-19): a cross-country analysis. J. Travel Res. 60, 687–692. doi: 10.1177/0047287520931593

[ref32] Faul F. Erdfelder E. Buchner A. Lang A. G. (2009). Statistical power analyses using G*Power 3.1: tests for correlation and regression analyses. Behav. Res. Methods 41, 1149–1160. 19897823 10.3758/BRM.41.4.1149

[ref33] Faul F. Erdfelder E. Lang A. G. Buchner A. (2007). G*Power 3: a flexible statistical power analysis program for the social, behavioural, and biomedical sciences. Behav. Res. Methods 39, 175–191. 17695343 10.3758/bf03193146

[ref34] Faulkner J. O’Brien W. J. McGrane B. Wadsworth D. Batten J. Askew C. D.. (2021). Physical activity, mental health and well-being of adults during initial COVID-19 containment strategies: a multi-country cross-sectional analysis. J. Sci. Med. Sport 24, 320–326. doi: 10.1016/j.jsams.2020.11.016, PMID: 33341382 PMC7711171

[ref35] Ferdinand J. A. Schma W. Yates J. Hora P. Martin J. (2002). The judicial perspective. Fordham Urban Law J. 29:2011.

[ref36] Flynn B. B. Sakakibara S. Schroeder R. G. Bates K. A. Flynn E. J. (1990). Empirical research methods in operations management. J. Oper. Manag. 9, 250–284. doi: 10.1016/0272-6963(90)90098-X, PMID: 36624513

[ref37] Foo L. P. Chin M. Y. Tan K. L. Phuah K. T. (2020). The impact of COVID-19 on the tourism industry in Malaysia. Curr. Issue Tour., 1–5. doi: 10.1080/13683500.2020.1777951

[ref38] Fu Y. Y. Mount D. J. (2002). Older workers’ communication satisfaction in the lodging industry. J. Hum. Resour. Hospital. Tour. 1, 59–73. doi: 10.1300/J171v01n01_05

[ref39] Fuchs G. Reichel A. (2011). An exploratory inquiry into destination risk perceptions and risk reduction strategies of first time vs. repeat visitors to a highly volatile destination. Tour. Manag. 32, 266–276. doi: 10.1016/j.tourman.2010.01.012

[ref40] Grönroos C. (1984). A service quality model and its marketing implications. Eur. J. Mark. doi: 10.1108/EUM0000000004784, PMID: 36554447

[ref41] Hair J. F. Black W. C. Babin B. J. Anderson R. E. (2010). Multivariate Data Analysis. In *Pearson Custom Library*. doi: 10.1038/259433b0

[ref42] Hair J. F. Black W. C. Babin B. J. Anderson R. E. Tatham R. L. (2006). Multivariate data analysis. In *Pearson Prentice Hall*. 6. doi: 10.1080/19447013008687143

[ref9004] Halme J. (2021). The role of social capital in the institutionalization of regional place marketing activity. Place Branding and Public Diplomacy 17, 249–256.

[ref43] Hampson D. P. McGoldrick P. J. (2013). A typology of adaptive shopping patterns in recession. J. Bus. Res. 66, 831–838. doi: 10.1016/j.jbusres.2011.06.008

[ref44] Hermiyenti S. Wardi Y. (2019). A literature review on the influence of promotion, price and brand image to purchase decisions. 2nd Padang International Conference on Education, Economics, Business and Accounting (PICEEBA-2 2018) (pp. 254–261). Atlantis Press.

[ref45] Ibrahim A. U. Daniel C. O. (2019). Impact of leadership on organizational performance. Int. J. Bus. Manag. Soc. Res. 6, 367–374. doi: 10.18801/ijbmsr.060219.39, PMID: 36612690

[ref46] Jaakkola E. (2007). Purchase decision-making within professional consumer services: organizational or consumer buying behaviour? Mark. Theory 7, 93–108. doi: 10.1177/1470593107073847, PMID: 36620800

[ref47] Jayanti R. D. Zuhri M. Z. (2017). Analisis Pengaruh Iklan Dan Harga Terhadap Keputusan Pembelian Minuman Teh Pucuk Harum Pada Konsumen De Nala Foodcourt. Eksis 12. doi: 10.26533/eksis.v12i1.78

[ref48] Jeaheng Y. Al-Ansi A. Han H. (2020). Impacts of halal-friendly services, facilities, and food and beverages on Muslim travellers' perceptions of service quality attributes, perceived price, satisfaction, trust, and loyalty. J. Hosp. Market. Manag. 29, 787–811. doi: 10.1080/19368623.2020.1715317

[ref49] Jiang Y. Wen J. (2020). Effects of COVID-19 on hotel marketing and management: a perspective article. Int. J. Contemp. Hosp. Manag. doi: 10.1108/IJCHM-03-2020-0237, PMID: 36415127

[ref50] Kaushal V. Srivastava S. (2021). Hospitality and tourism industry amid COVID-19 pandemic: perspectives on challenges and learnings from India. Int. J. Hosp. Manag. 92:102707. doi: 10.1016/j.ijhm.2020.102707, PMID: 33024348 PMC7528873

[ref51] Kim H. Y. (2013). Statistical notes for clinical researchers: assessing normal distribution (2) using skewness and kurtosis. Restor. Dent. Endod. 38, 52–54. doi: 10.5395/rde.2013.38.1.52, PMID: 23495371 PMC3591587

[ref52] Köksal M. S. Ertekin P. Çolakoğlu Ö. M. (2014). How are differences among data collectors reflected in the reliability and validity of data collected by LikertType scales? Educat. Sci. 14. doi: 10.12738/estp.2014.6.2028

[ref53] Kotler P. Armstrong G. (1996). Principles of Marketing. Prentice Hall, Englewood Cliffs, NJ.

[ref54] Kotler P. Keller K. L. (2012). Marketing Management: Philip Kotler, Kevin Lane Keller, Pearson. Upper Saddle River, NJ.

[ref55] Kotler P. Keller K. L. (2016). A Framework for Marketing Management. 352 Boston, MA: Pearson.

[ref56] Kuspriyono T. Nurelasari E. (2018). Pengaruh social media marketing terhadap customer bonding dan purchase to intention. Cakrawala-Jurnal Humaniora 18, 235–242.

[ref57] Laksmi M. S. Bagia W. Trianasari N. (2022). The effect of peer influence and service quality on insurance product purchase decisions (case study of Pt. Asuransi Jiwa Sinarmas MSIG Tbk Singaraja branch). Int. J. Soc. Sci. Bus. 6. doi: 10.23887/ijssb.v6i1.44034

[ref58] Lepp A. Gibson H. (2003). Tourist roles, perceived risk and international tourism. Ann. Tour. Res. 30, 606–624. doi: 10.1016/S0160-7383(03)00024-0, PMID: 36148117

[ref59] Lepp A. Gibson H. Lane C. (2011). Image and perceived risk: a study of Uganda and its official tourism website. Tour. Manag. 32, 675–684. doi: 10.1016/j.tourman.2010.05.024

[ref60] Lien P. T. (2017). Factors affecting lecturer job satisfaction: case of Vietnam universities. Int. J. Acad. Res. Econom. Manag. Sci. 6, 138–148.

[ref61] Liu Y. Jang S. S. (2009). Perceptions of Chinese restaurants in the US: what affects customer satisfaction and behavioral intentions? Int. J. Hosp. Manag. 28, 338–348. doi: 10.1016/j.ijhm.2008.10.008

[ref62] Malaysian Association of Hotels [MAH]. (2020). Tourism comes to a standstill. Available at: https://www.hotels.org.my/press/22578-tourism-comes-to-a-standstill

[ref63] Mattila A. S. (2006). How effective commitment boosts guest loyalty (and promotes frequent-guest programs). Cornell Hotel Restaur. Admin. Q. 47, 174–181. doi: 10.1177/0010880405283943

[ref64] McKenzie D. Schargrodsky E. Cruces G. (2011). Buying less but shopping more: the use of nonmarket labour during a crisis [with comment]. Economia 11, 1–43.

[ref65] Ministry of Finance (2020). 2020 economic stimulus package “bolstering confidence, stimulating growth & protecting jobs.” Available at: https://www.treasury.gov.my/pdf/pre2020/speech_2020_Economic_Stimulus_Package.pdf

[ref9005] Mitchell V. W. (1992). Understanding consumers’ behaviour: can perceived risk theory help?. Management decision.

[ref9006] Mitchell V. W. Vassos V. (1998). Perceived risk and risk reduction in holiday purchases: A cross-cultural and gender analysis. Journal of Euromarketing 6, 47–79.

[ref66] Nasikan S. A. (2013). Faktor Internal dan Eksternal Terhadap Keputusan Pembelian Telepon Selular Merk Nokia. Jurnal: Manajemen dan Akuntansi:2.

[ref67] Nurcahyo R. Fitriyani A. Hudda I. N. (2017). The influence of facility and service quality on customer satisfaction and its impact on customer loyalty in Borobudur Hotel in Jakarta. BINUS Bus. Rev. 8, 23–29.

[ref68] O’Neill M. Palmer A. (2001). Survey timing and consumer perceptions of service quality: an overview of empirical evidence. Manag. Serv. Qual. doi: 10.1108/09604520110391351

[ref69] Oliver R. L. (1980). A cognitive model of the antecedents and consequences of satisfaction decisions. J. Mark. Res. 17, 460–469. doi: 10.1177/002224378001700405, PMID: 23326940

[ref70] Parasuraman A. Zeithaml V. A. Berry L. L. (1985). A conceptual model of service quality and its implications for future research. J. Mark. 49, 41–50. doi: 10.1177/002224298504900403, PMID: 36535910

[ref71] Petrenko A. Ekinil G. Provotorina V. Davidova E. (2021). “Development of modern forms of hotel farms in the region” in E3S Web of Conferences, Vol. 273 (EDP Sciences), 09017.

[ref72] Pratiwi A. P. Murwanti S. (2017). Pengaruh service quality dan promotion Terhadap Intens to repurchase Jasa service motor Dengan customer satisfaction Sebagai intervening variable (Studi pada Bengkel motor Ahass Cabang UMS). Doctoral dissertation Universitas Muhammadiyah Surakarta.

[ref74] Raja Omar R. N. Nik Hashim N. A. A. Zain E. N. M. Ramlee S. I. F. Abdul Halim A. F. Mohd Rohzi A. F.. (2020). Factors that influence online behavior in purchasing hotel room via website among tourist. Eur. J. Mol. Clin. Med.

[ref75] Ramadhani R. Rofiqul U. M. A. M. Abdurrahman A. Syazali M. (2019). The effect of flipped-problem-based learning model integrated with LMS-google classroom for senior high school students. J. Educat. Gifted Young Scient. 7, 137–158. doi: 10.17478/jegys.548350, PMID: 35789610

[ref76] Răvar A. (2011). The importance of print and visual media in the promotion of hospitality enterprises. Cactus Tour. J. 2, 93–98.

[ref77] Rehman A. Liu G. Yousaf B. Zia-ur-Rehman M. Ali M. U. Rashid M. S.. (2020). Characterizing pollution indices and children health risk assessment of potentially toxic metal(oid)s in school dust of Lahore, Pakistan. Ecotoxicol. Environ. Saf. 190:110059. doi: 10.1016/j.ecoenv.2019.110059, PMID: 31837569

[ref78] Reisinger Y. Mavondo F. (2005). Travel anxiety and intentions to travel internationally: implications of travel risk perception. J. Travel Res. 43, 212–225. doi: 10.1177/0047287504272017

[ref80] Sao A. Singh S. Dixit S. Pandey A. K. Singh S. (2017). Quality, productivity and customer satisfaction in service operations: an empirical study. Int. J. Mech. Eng. Technol. 8, 579–596.

[ref81] Setiawan H. Sayuti A. J. (2017). Effects of service quality, customer trust and corporate image on customer satisfaction and loyalty: an assessment of travel agencies customer in South Sumatra Indonesia. IOSR J. Bus. Manag. 19, 31–40.

[ref82] Shafiee M. M. Bazargan N. A. (2018). Behavioural customer loyalty in online shopping: the role of e-service quality and e-recovery. J. Theor. Appl. Electron. Commer. Res. 13, 26–38. doi: 10.4067/S0718-18762018000100103

[ref83] Shah K. Kamrai D. Mekala H. Mann B. Desai K. Patel R. S. (2020). Focus on mental health during the coronavirus (COVID-19) pandemic: applying learnings from the past outbreaks. Cureus 12. doi: 10.7759/cureus.7405, PMID: 32337131 PMC7182052

[ref84] Shah A. U. M. Safri S. N. A. Thevadas R. Noordin N. K. Rahman A. A. Sekawi Z.. (2020). COVID-19 outbreak in Malaysia: actions taken by the Malaysian government. Int. J. Infect. Dis. 97, 108–116. doi: 10.1016/j.ijid.2020.05.093, PMID: 32497808 PMC7264933

[ref85] Shin H. Kang J. (2020). Reducing perceived health risk to attract hotel customers in the COVID-19 pandemic era: focused on technology innovation for social distancing and cleanliness. Int. J. Hosp. Manag. 91:102664. doi: 10.1016/j.ijhm.2020.102664, PMID: 32921871 PMC7476579

[ref86] The Star. (2020). Hoteliers: the hospitality industry, among the worst hit by the pandemic, has lost RM11.3bil to date | the star. Available at the star digital publication website: https://www.thestar.com.my/news/nation/2021/06/25/hoteliers-hospitality-industry-among-the-worst-hit-by-pandemic-has-lost-rm113bil-to-date (Accessed July 4, 2021).

[ref87] Uğur N. G. Akbıyık A. (2020). Impacts of COVID-19 on global tourism industry: a cross-regional comparison. Tour. Manag. Perspect. 36:100744. doi: 10.1016/j.tmp.2020.100744 32923356 PMC7474895

[ref88] Vilnai-Yavetz I. Gilboa S. (2010). The effect of servicescape cleanliness on customer reactions. Serv. Mark. Q. 31, 213–234. doi: 10.1080/15332961003604386

[ref89] Vlontzos G. Duquenne M. N. (2014). Assess the impact of subjective norms on consumers' behaviour in the Greek olive oil market. J. Retail. Consum. Serv. 21, 148–157. doi: 10.1016/j.jretconser.2013.09.003

[ref90] Williams A. M. Baláž V. (2015). Tourism risk and uncertainty: theoretical reflections. J. Travel Res. 54, 271–287. doi: 10.1177/0047287514523334

[ref91] Wiyadi W. Ayuningtyas N. A. (2019). Product aspects of marketing effort and purchase intention. Humanit. Soc. Sci. Rev. 7, 541–547. doi: 10.18510/hssr.2019.7380, PMID: 14965061

[ref92] Wongleedee K. (2015). Marketing mix and purchasing behaviour for community products at traditional markets. Procedia Soc. Behav. Sci. 197, 2080–2085. doi: 10.1016/j.sbspro.2015.07.323

[ref93] World Health Organisation [WHO]. (2020). Coronavirus disease (COVID-19). Available at World Health Organization website: https://www.who.int/emergencies/diseases/novel-coronavirus-2019?gclid=CjwKCAjwhYOFBhBkEiwASF3KGWEBMdtOoR95DWOy1fuyDlSAj9wQaPUxaOQeInW5H6FlYlZKZxYPRxoCOdYQAvD_BwE (Accessed May 17, 2021).

[ref95] Yang C. L. Nair V. (2014). Risk perception study in tourism: are we really measuring perceived risk? Procedia Soc. Behav. Sci. 144, 322–327. doi: 10.1016/j.sbspro.2014.07.302, PMID: 36429599

[ref96] Yoe C. (2019). Principles of risk analysis: Decision making under uncertainty CRC Press.

[ref97] Zemke D. M. V. Neal J. Shoemaker S. Kirsch K. (2015). Hotel cleanliness: will guests pay for enhanced disinfection? Int. J. Contemp. Hosp. Manag. 27, 690–710. doi: 10.1108/IJCHM-01-2014-0020

[ref98] Zeng L. Xia S. Yuan W. Yan K. Xiao F. Shao J.. (2020). Neonatal early-onset infection with SARS-CoV-2 in 33 neonates born to mothers with COVID-19 in Wuhan, China. JAMA Pediatrics 174, 722–725. doi: 10.1001/jamapediatrics.2020.0878, PMID: 32215598 PMC7099530

[ref9007] Zietsman M. L. Mostert P. Svensson G. (2018). Perceived price and service quality as mediators between price fairness and perceived value in business banking relationships: A micro-enterprise perspective. International Journal of Bank Marketing 37, 2–19.

